# An FIA-MS Method for Rapid Coffee Adulteration Detection: A Comparative Study with a Non-Targeted LC-MS Approach

**DOI:** 10.3390/foods14172931

**Published:** 2025-08-22

**Authors:** Nerea Núñez, Javier Saurina, Oscar Núñez

**Affiliations:** 1 Department of Chemical Engineering and Analytical Chemistry, University of Barcelona, Martí i Franquès 1-11, E08028 Barcelona, Spain; xavi.saurina@ub.edu (J.S.); oscar.nunez@ub.edu (O.N.); 2 Research Institute in Food Nutrition and Food Safety, University of Barcelona, Recinte Torribera, Av. Prat de la Riba 171, Edifici de Recerca (Gaudí), Santa Coloma de Gramenet, E08921 Barcelona, Spain; 3 Serra Húnter Fellow, Departament de Recerca i Universitats, Generalitat de Catalunya, Via Laietana 2, E08003 Barcelona, Spain

**Keywords:** flow injection analysis–mass spectrometry (FIA-MS), rapid analysis, coffee authentication, food adulteration, chemometrics

## Abstract

Coffee adulteration is a growing concern in the food industry due to economic and quality implications. This study evaluates a rapid, non-targeted fingerprinting method based on flow injection analysis–mass spectrometry (FIA-MS) for detecting common coffee adulterants. A total of 119 samples were analyzed, including 43 coffee samples and 76 samples of common coffee adulterants (16 chicory, 10 barley, and 50 flour samples). FIA-MS combined with chemometric analysis allowed for the classification of pure and adulterated coffee samples with over 95% accuracy. Compared to LC-MS, the FIA-MS method showed a similar performance while offering significantly faster analysis and lower solvent consumption, making it a practical and sustainable option for high-throughput screening. For PLS regression studies, calibration and prediction errors were consistently below 0.91% and 11.7%, respectively. Furthermore, the methodology was compared with a non-targeted LC-MS approach, showing an excellent performance.

## 1. Introduction

Coffee, one of the most popular and widely consumed beverages globally, plays a vital role in the global economy, particularly in countries that rely on its production and export. Its appeal extends beyond its distinctive aroma and taste, as it also possesses bioactive properties associated with health benefits, such as antioxidant activity, thanks to compounds like phenolic acids, polyphenols, and alkaloids. However, this widespread popularity has made coffee a common target for fraud and adulteration, practices aimed at maximizing economic profits at the expense of product quality and safety and consumer trust [1,2,3,4].

Coffee adulteration can take various forms. Commonly, lower-value coffee beans, such as Robusta, are added to blends labeled as 100% Arabica, misleading consumers about the quality of the product. In other cases, unrelated substitutes, such as chicory, barley, corn, rice, and various flours, are introduced, altering both the chemical composition and organoleptic properties of the coffee. These additives can significantly change the sensory and nutritional profile of the product, affecting its authenticity. For instance, chicory, which has historically been used in certain regions as a coffee complement, imparts distinctive flavors and alters the concentration of essential bioactive compounds. Similarly, barley and flour introduce carbohydrates and proteins that are absent in pure coffee, affecting its perceived quality [5,6,7].

Beyond the economic deception, adulteration poses health risks, especially when added materials contain allergens or contaminants. Detecting these practices is challenging, as adulterants are often present in small quantities and do not visibly alter the appearance of the product, yet they impact its molecular profile and functionality. This presents a challenge for both regulators and producers, who must ensure their products meet the quality standards set by international regulations [5,8,9,10].

In this context, the development of advanced analytical techniques has become crucial for detecting and preventing adulterations in coffee. Traditional methodologies such as liquid chromatography (LC) or gas chromatography (GC) coupled to mass spectrometry (MS) have proven effective [7,11]. LC-based methods often involve reversed-phase separations followed by ultraviolet (UV), fluorescence (FL), or mass spectrometry (MS) detection. For example, Bressanello et al. [12] employed an HPLC-UV non-targeted method to analyze non-volatile compounds in coffee, with the aim of identifying chemical patterns associated with taste attributes. In another work, Wan et al. [13] applied an HPLC-FLD method to determine EU-priority PAHs in coffee samples, aiming to evaluate how the roasting degree and brewing methods influence PAH levels and their transfer into the final beverage. In another example, Núñez et al. [14] used untargeted HPLC-UV-FLD fingerprinting combined with chemometric analysis to evaluate coffee authenticity and detect adulteration with common additives, such as chicory, barley, and various flours. Moreover, LC-MS techniques are still widely used for the comprehensive analysis of coffee-related compounds [7,11]. For instance, Artêncio et al. [15] proposed a UHPLC-Orbitrap HRMS method for the non-targeted analysis of Brazilian coffee, using a C18 column for chromatographic separation and collecting full-scan data in both positive and negative ESI modes over an *m*/*z* window of 100–1500. Alternatively, GC-based methods usually employ MS detection [16]. For example, Núñez et al. [17] utilized HS-SPME-GC-MS with chemometric data analysis to characterize and classify coffee samples, including different coffee varieties and origins, chicory, and soluble coffees, based on their volatile compound profiles.

These methods offer excellent analytical performances; however, they are often hindered by drawbacks such as long analysis times, high operational costs, extensive sample preparation, or limited suitability for on-site or high-throughput environments. In this context, more agile methods are being explored, such as flow injection analysis coupled with mass spectrometry (FIA-MS), which allows for the detection of adulterants with high sensitivity in significantly less time. This technique eliminates the need for prior chromatographic separation, making it ideal for analyzing large volumes of samples. FIA-MS is particularly advantageous for quality control in the coffee industry, as it provides rapid and accurate results without compromising sensitivity or specificity. Furthermore, although portable NIR or GC-IMS instruments allow for rapid in-field screening, FIA-MS provides a laboratory-based, high-throughput alternative that combines speed with enhanced chemical selectivity and sensitivity, which is particularly valuable for the detection of complex coffee adulterations [18,19].

Several recent studies have focused on the use of FIA-MS for food analysis, illustrating its growing relevance and effectiveness in this area. For instance, Campmajó et al. [20] explored the potential of FIA-HRMS fingerprinting for food authentication, particularly for distinguishing between red wine, paprika, and vegetable oils. This approach, which was combined with chemometrics, demonstrated excellent classification accuracies when used to assess the geographical origins and quality categories of the food products. Furthermore, the study by Vilà et al. [21] highlighted the power of FIA-MS fingerprinting in differentiating between various types of tea and detecting adulterants like chicory. In a similar vein, Zhang et al. [22] demonstrated the FIA-MS/MS effectiveness in quantifying ochratoxin A in food samples like corn and grape juice. FIA-MS/MS showed comparable results to those of traditional LC-MS/MS techniques, but with a significantly reduced analysis time. In a more specific application, Calvarro et al. [23] employed FIA to measure the fluorescent compounds formed during the Maillard reaction in cookies. These works demonstrated the potential of FIA-MS for rapid screening and classification in food matrices such as tea. However, its application to coffee authentication and adulteration detection remains largely unexplored. The present study expands this application to roasted coffee, a matrix with greater chemical complexity due to roasting and a higher risk of economic adulteration, thereby testing the method under more challenging conditions.

The aim of this work was to develop and validate a rapid, efficient, and robust analytical strategy based on FIA-MS fingerprinting, and to comprehensively compare its classification performance, greenness, and practical applicability with a conventional LC-MS approach for the authentication of roasted coffee. The proposed approach is based on non-targeted chemical fingerprinting using FIA-MS, offering high-throughput capabilities. To complement and reinforce the accuracy and reproducibility of the results, a parallel methodology using LC coupled to a QTRAP mass spectrometer (LC-MS) was also implemented.

## 2. Materials and Methods

### 2.1. Chemicals and Solutions

HPLC-grade methanol (Chromosolv™, ≥99.9%) suitable for HPLC analyses was procured from PanReac AppliChem (Barcelona, Spain). Formic acid (≥98%) was obtained from Sigma-Aldrich (St. Louis, MO, USA). Ultrapure water was produced using an Elix 3 system coupled to a Milli-Q purification unit (Millipore, Bedford, MA, USA), equipped with an integrated 0.22 µm membrane of nylon filter to ensure particle removal.

### 2.2. Samples

As described in Table S1 (in the Supplementary Materials), 119 samples were analyzed. The dataset comprised 43 coffee samples, including both Arabica and Robusta varieties, as well as blends, sourced from various Vietnamese and Cambodian coffees. To simulate diverse adulteration scenarios, 76 samples of common coffee adulterants were also included, comprising 16 chicory samples from different commercial brands, 50 flour samples encompassing wheat, rice, rye, cornmeal, and oatmeal flours from various organic and integral product lines, and 10 barley samples covering both malted and pearled forms. This carefully selected sample set was designed to reflect realistic and varied cases of coffee adulteration encountered in the market.

To evaluate the robustness of the chemometric models and the repeatability of the analytical procedures, a quality control (QC) solution was prepared by mixing 50 μL from each of the 119 extracts (including both genuine coffees and adulterated samples). This QC solution resulted in a pooled sample representative of the overall chemical variability. This QC was used to monitor the signal consistency and instrument performance throughout the analytical sequence.

The adulteration studies were performed using Arabica coffee samples from Vietnam and chicory, barley, and flour as the adulterants. Three different adulteration scenarios were evaluated: Vietnamese Arabica vs. chicory, Vietnamese Arabica vs. flour, and Vietnamese Robusta vs. barley. In each case, the calibration set consisted of mixtures prepared at adulteration levels of 20%, 40%, 60%, and 80%, together with the corresponding 100% pure coffee samples, as shown in Table S2 (in the Supplementary Materials). The validation set included mixtures at adulteration levels of 15%, 25%, 50%, 75%, and 85%. All adulterated mixtures were prepared in quintuplicate, resulting in a total of 55 sample extracts per case. Furthermore, a sample adulterated at 50% concentration was employed as the QC for each type of system.

### 2.3. Sample Treatment

Coffee and adulterant samples that were already in powdered form were used directly. Whole-bean samples were ground using a coffee grinder prior to extraction.

To accurately simulate realistic scenarios of coffee adulteration, all barley and flour samples were subjected to a roasting process before extraction. For this, 80.00 g of each sample was spread evenly on a stainless-steel tray and roasted in a conventional oven (Teka HE 510 Me, Barcelona, Spain) for 7 min at 180 °C. The sample layer was kept consistent at an approximately 5 mm thickness to ensure reproducible heating conditions across all batches.

For the extraction, 1.00 g, accurately weighed, of each sample was placed into a 15 mL PTFE centrifuge tube (Serviquimia, Barcelona, Spain), followed by the addition of 10 mL of a 50:50 (*v*/*v*) methanol–water mixture. The resulting suspension was vortexed for 2 min using a Vortex mixer (Stuart, Stone, UK). Subsequently, the sample was centrifuged at 3500 rpm for 5 min with a Rotanta 460 RS centrifuge (Hettich, Tuttlingen, Germany). The resulting aqueous methanolic extracts were then filtered through 0.45 μm nylon filters (discarding the first mL) into injection vials, and the filtered extracts were stored at −4 °C until analysis (within 48 h to ensure stability and minimize degradation).

### 2.4. Instrumentation

The analysis of the coffee samples was carried out using an Agilent 1100 Series liquid chromatograph (Agilent Technologies, Palo Alto, CA, USA) with a vacuum degasser (model G1322A), a binary pump (model G1312A), and an autosampler (model G1367A). This chromatographic system was coupled with an Applied Biosystems 4000 QTrap hybrid triple quadrupole/linear ion trap mass spectrometer (AB Sciex, Framingham, MA, USA) for both FIA-MS and LC-MS studies. Data acquisition and instrument control were managed using Analyst software version 1.7.3.

The FIA-MS involved direct injection of 5 µL of sample into a carrier solution of 0.1% formic acid (*v*/*v*) at a steady flow rate of 150 µL/min, allowing for rapid analysis with a total runtime of 1.5 min and 144 scan cycles over the entire FIA peak. Data acquisition was performed in negative electrospray ionization (ESI) mode using enhanced mass spectrometry (EMS) scan mode over an *m*/*z* range of 100–550, summing two scans per spectrum. The scan rate was faster at 4000 Da/s.

For the LC-MS method, chromatographic separation was carried out on a Kinetex^®^ C18 reversed-phase column (100 mm × 4.6 mm, 2.6 µm particle size) manufactured by Phenomenex, Torrance, CA, USA. The mobile phase consisted of ultrapure water with 0.1% formic acid (*v*/*v*) (solvent A) and methanol (solvent B), delivered at a flow rate of 0.4 mL/min. The gradient elution began at 3% solvent B, increasing linearly to 75% over 30 min, followed by an increase to 95% between 30 and 32 min, held constant for 2 min, and then returned to initial conditions with a 6 min equilibration. Data acquisition was performed in negative ESI mode, as indicated in the FIA-MS method, with a total analysis time of 40 min comprising 1837 scan cycles over the entire chromatogram.

In the two methods, all samples were randomly analyzed to minimize the influence of instrumental drifts; quality control (QC) samples (see Section 2.2) were injected periodically every ten samples to assess the reproducibility throughout the analysis.

### 2.5. Data Preprocessing

Data obtained from the FIA-MS and LC-MS/QTRAP techniques were processed as described below. To simplify the dataset, an absolute intensity threshold of 10^4^ was applied using the open-source software MSConvert v3.0. The resulting filtered files were then analyzed with MZmine-2.53, generating Excel spreadsheets containing identified chemical features, including ion signals linked to *m*/*z* values (for both techniques) and retention times (specific to LC-MS). During processing in MZmine-2.53, exact mass detection was performed to create a mass list of individual ions for each MS spectrum across the dataset, applying a noise cutoff at 1 × 10^4^. Finally, all mass lists were curated to remove remaining artefacts by applying the FTMS shoulder peak filter. Following this, the parameters, including an FIA peak time range of 0.05–2 min (0.05—39.95 min in the case of LC-MS), an *m*/*z* tolerance of 5 ppm, and an intensity threshold of 2.0 × 10^4^, were established. This range was selected based on the total ion chromatograms (TICs) across all samples and included the full FIA peak, from the injection front to the tailing zone, while excluding baseline regions.

In the LC-MS analysis, the chromatogram builder method was used to align exact mass signals detected in consecutive scans of each sample. Chromatogram deconvolution was then carried out to separate individual peaks within each detected fingerprint. The join aligner tool matched the exact masses found across the samples (using a mass tolerance of 5 ppm) with their respective retention times (allowing a tolerance of 2.5 min).

Ion signal intensity data from both FIA-MS and LC-MS were exported as Excel files, producing a data matrix structured by samples versus variables, where the variables included the *m*/*z* values and, for LC-MS, the chromatographic retention times. The resulting chemical fingerprints were filtered to remove sporadic features that appeared in only a few samples and did not represent consistent patterns; only features detected in at least five samples were kept. In this study, a chemical fingerprint is the pattern of ion signals defined by their *m*/*z* ratio and intensity, along with retention time for LC-MS, obtained from the mass spectrometry data of a single sample. It represents the chemical profile of that sample without identifying specific compounds and is used to compare and classify samples.

### 2.6. Data Analysis

The final matrices, containing 699 features for FIA-MS and 10,288 features for LC-MS, were subsequently utilized for PCA, PLS-DA, and PLS using SOLO 8.6 software from Eigenvector Research (Manson, WA, USA) [24]. The theoretical underpinnings of these chemometric methodologies are discussed elsewhere [25].

For every employed methodology, the X-data matrices prepared for PCA and PLS-DA comprised the metabolomic information corresponding to the analyzed samples and QCs. In all instances, a normalization pretreatment was applied to ensure similar weights across all samples using the closest QC as the reference. The Y-data in the PLS-DA defined the class membership of each coffee sample. Scatter plots of scores derived from the principal components (PCs) were generated to evaluate the robustness of the employed methodology and the classification patterns exhibited by the samples. For the PLS-DA, scatter plots depicting scores from latent variables (LVs) were utilized to analyze the distribution of samples.

Paired PLS-DA models were developed and validated using independent prediction sets. To this end, the PLS-DA calibration used 70% of the randomly selected sample group, and the remaining 30% constituted the prediction set.

For the PLS models, validation was performed using prediction sets with adulteration levels of 15%, 25%, 50%, 75%, and 85%, as outlined in Section 2.2.

The optimal number of LVs for both PLS-DA and PLS was determined as the first significant minimum point of cross-validation (CV) errors following a Venetian blind strategy.

## 3. Results and Discussion

### 3.1. High-Throughput FIA-MS and LC-MS Fingerprints

The main objective of this study was to investigate whether chemical fingerprints, obtained by FIA-MS and LC-MS, could be effectively used to discriminate between pure coffee samples and common adulterants, as well as to differentiate coffee types based on their variety and geographical origin. The FIA-MS and LC-MS methodologies were operated under negative ESI, covering a *m*/*z* range of 100 to 550.

**Figure 1:**
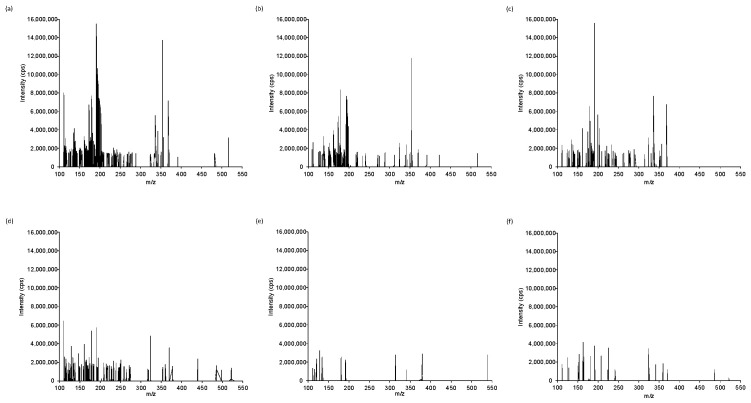
Non-targeted FIA-MS fingerprints for selected (**a**) Vietnamese Arabica coffee, (**b**) Vietnamese Robusta coffee, (**c**) Cambodian coffee, (**d**) chicory, (**e**) flour, and (**f**) barley.

Sample extracts, prepared as described in Section 2.3, were analyzed by both FIA-MS and LC-MS to generate chemical fingerprints without compound-specific identification. As representative examples, Figure 1 and Figure S1 (in the Supplementary Materials) show the chemical fingerprints of selected samples (Vietnamese Arabica and Robusta coffee, Cambodian coffee, chicory, wheat flour, and barley) obtained by FIA-MS and LC-MS, respectively. These fingerprints correspond to the peak intensities of the extracted ion features at each *m*/*z* value obtained after Mzmine processing. Each plot displays the *m*/*z* values (*x*-axis) and their corresponding signal intensities (*y*-axis, expressed as peak areas) for one representative sample.

In the FIA-MS dataset (Figure 1), the most intense signals were concentrated in the low-*m*/*z* region (100–250). For instance, the ion at *m*/*z* 111.18 was highly intense in Vietnamese Arabica and Robusta coffees but absent or very weak in chicory and flour samples, while chicory showed prominent signals at *m*/*z* 113.14 and 115.03, which were considerably less intense in the coffee profiles. Barley presented a distinct signal at *m/z* 113.10, which was also detected in coffee samples but with markedly different relative intensities. Despite the absence of chromatographic separation, the FIA-MS fingerprints were highly reproducible and sufficiently specific for each matrix. Signals in the medium- and high-*m*/*z* ranges (*m*/*z* > 250) were also observed, although they appeared less frequently and with lower intensities. The rapid acquisition time (~1.5 min per sample) further highlights the suitability of FIA-MS for high-throughput screening purposes.

In contrast, the LC-MS profiles (Figure S1) incorporated reversed-phase chromatographic separation, which provided an additional temporal dimension and higher spectral resolution. Although the *m/z* range was identical to that of the FIA-MS, the LC-MS data revealed a broader distribution of signals across the entire spectrum, including medium (*m*/*z* 250–400) and high (*m*/*z* > 400) regions, where well-defined peaks were observed in several matrices. Coffee samples displayed complex and distributed fingerprints, while adulterants such as flour and chicory showed more discrete and isolated peaks. The enhanced separation enabled improved discrimination, even between samples with overlapping spectral patterns. Marker ions such as *m*/*z* 111.18 and 113.14 were identified as useful features for distinguishing coffee from non-coffee matrices in both techniques, with their retention time behavior in LC-MS contributing to increased specificity.

In summary, FIA-MS and LC-MS offer different, but complementary, advantages. FIA-MS enables the rapid acquisition of high-throughput feature-based fingerprints, making it suitable for the high-throughput screening of large sample sets. Thus, the use of both techniques enables the generation of reliable chemical descriptors for sample classification and authentication.

### 3.2. PCA Exploration

PCA was employed as an exploratory tool to evaluate the ability of the FIA-MS and LC-MS data to capture the main sources of chemical variability among the studied coffee samples and adulterants. Autoscaling was applied to the ion intensity values to ensure the equal contribution of all variables. Additionally, a QC-based normalization strategy was applied to correct for instrumental drift over time: for each feature, the peak area intensity of a given sample was divided by the corresponding value in the nearest QC sample. This approach allowed for signal correction throughout the sequence, improving the clustering structure observed in multivariate models. Hence, this normalization procedure stabilized the overall dataset and enhanced the interpretability of the results.

**Figure 2:**
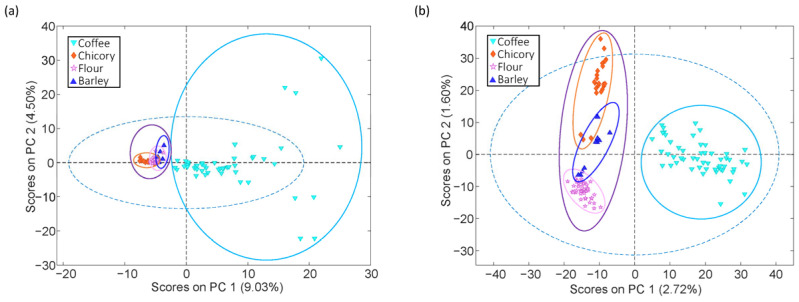
PCA score plots obtained using (**a**) FIA-MS and (**b**) LC-MS data to study coffee samples in front of chicory, flour, and barley adulterants.

Figure 2 shows the PCA score plots obtained using (a) FIA-MS and (b) LC-MS data. Although the percentages of variance explained by PCs are low, this is expected in non-targeted MS fingerprinting studies, where data dimensionality is high and many variables contribute modestly to the total variance. However, the PCA projections still reveal class-related trends, supporting their usefulness for exploratory purposes. In both cases, a clear clustering pattern is observed, with coffee samples distinctly separated from each of the adulterants along the first principal component (PC1). In the case of FIA-MS data (Figure 2a), the separation of pure coffee samples from adulterants indicates remarkable chemical differences, as captured by the FIA-MS fingerprint. Similarly, the PCA model built using LC-MS data (Figure 2b) shows a comparable clustering pattern. Once again, coffee and adulterant samples occupy well-separated regions in the score plot. Notably, although the main objective was to differentiate coffee from its potential adulterants, the results also highlight that chicory, flour, and barley form compact and well-defined individual clusters, further emphasizing the distinct chemical profiles captured by the method. Coffee samples, in contrast, are positioned on the opposite side of the plot, reinforcing the effectiveness of the model for discriminative analysis.

The absence of overlap between coffee and adulterants supports the discriminative capacity of the non-targeted chemical descriptors. These results confirm that both the FIA-MS and LC-MS methodologies provide robust and informative chemical fingerprints that can distinguish authentic coffee samples from common non-coffee additives without needing prior compound identification.

Furthermore, taking advantage of the availability of coffee samples from different varieties (Arabica and Robusta) and geographical origins (Cambodia and Vietnam), a complementary study was conducted to evaluate the distribution of samples according to these attributes. The clustering results with FIA-MS and LC-MS data are presented in Figure S2 of the Supplementary Materials. The score plots reveal clear grouping according to variety or origin, demonstrating that the proposed non-targeted fingerprinting strategies are also capable of capturing intrinsic chemical differences within coffee. These findings support the applicability of FIA-MS and LC-MS data not only for detecting adulteration but also for coffee authentication based on variety and geographical origin.

### 3.3. PLS-DA for Classification of Coffee and Adulterant Samples

Supervised PLS-DA was applied to the chemical fingerprints obtained via non-targeted FIA-MS and LC-MS to explore class discrimination based on subtle differences in chemical composition, focusing on the identification of coffee versus chicory, flour, and barley.

For each PLS-DA model, the X-data matrix comprised the fingerprints generated by each analytical technique, and the Y-data matrix coded the class membership of each sample. Autoscaling pretreatment was applied to normalize the contribution of each variable.

**Figure 3:**
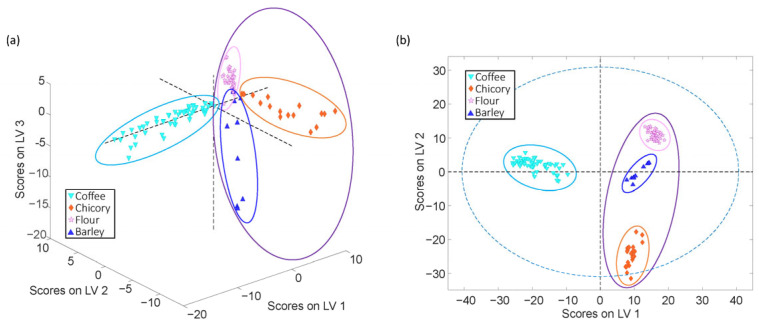
PLS-DA score plots for (**a**) FIA-MS and (**b**) LC-MS data to study coffee samples in front of chicory, flour, and barley adulterants.

Figure 3 shows the score plots resulting from the PLS-DA models for coffee *vs*. adulterant from the FIA-MS (Figure 3a) and LC-MS (Figure 3b) datasets. Clear visual separation between the coffee samples and the three adulterants is observed across both datasets, supporting the discriminative capacity of each analytical platform. The score plots reveal the distinct clustering of each sample class, not only differentiating coffee from the adulterants but also distinguishing between the different classes of adulterants themselves. Beyond this study, PLS-DA was also applied to classify the coffee samples according to variety (Arabica and Robusta) and geographical origin (Vietnam and Cambodia). The results of these classification models are presented in the Supplementary Materials (Figure S3). In the case of FIA-MS, Figure S3(a.1) shows an acceptable separation between Arabica and Robusta samples, while Figure S3(a.2) demonstrates a clear discrimination based on geographical origin. For LC-MS, an excellent classification performance was achieved, as shown in Figures S3(b.1) and S3(b.2), with well-defined clusters corresponding to both variety and origin, respectively.

Table S3 presents the performance metrics for each PLS-DA model, including the number of LVs and sensitivity, specificity, and classification error values. For the coffee vs. adulterant, three LVs were used. The FIA-MS-based models achieved excellent performances, with sensitivity and specificity higher than 87.5% and 90.8%, respectively, and classification errors lower than 6%. The LC-MS models outperformed the FIA-MS, achieving 100% values in sensitivity and specificity and 0% classification errors across all sample types.

Regarding the coffee variety, the FIA-MS and LC-MS models achieved sensitivity and specificity of 100%. Similarly, classification by geographical origin was successful with FIA-MS and LC-MS fingerprints, with 100% sensitivity and specificity values (see Table S3).

It is worth noting that while LC-MS provides a slightly superior classification performance, the FIA-MS platform offers significant advantages in terms of speed and simplicity. FIA-MS enables sample analysis in approximately 1.5 min (while the chromatographic run lasts 40 min). This makes FIA-MS an ideal choice for rapid screening applications, particularly in high-throughput environments, where slightly lower accuracy may be acceptable in exchange for faster turnaround. Moreover, it significantly reduces solvent use and reagent waste, making the method more environmentally sustainable and aligned with the principles of Green Analytical Chemistry and operational applicability assessments. This aspect will be further discussed in the greenness evaluation presented in Section 4.

### 3.4. PLS-DA Validation Through Paired Models

To assess the discrimination power of the FIA-MS and LC-MS methods, paired PLS-DA models were employed to classify the coffee samples against their common adulterants. Each model focused on binary comparisons between the coffee samples and a specific adulterant: chicory, flour, or barley. Additionally, paired comparisons were also conducted for the classification of the coffee samples by variety (Vietnamese Arabica vs. Vietnamese Robusta) and by geographical origin (Vietnam vs. Cambodia).

**Figure 4:**
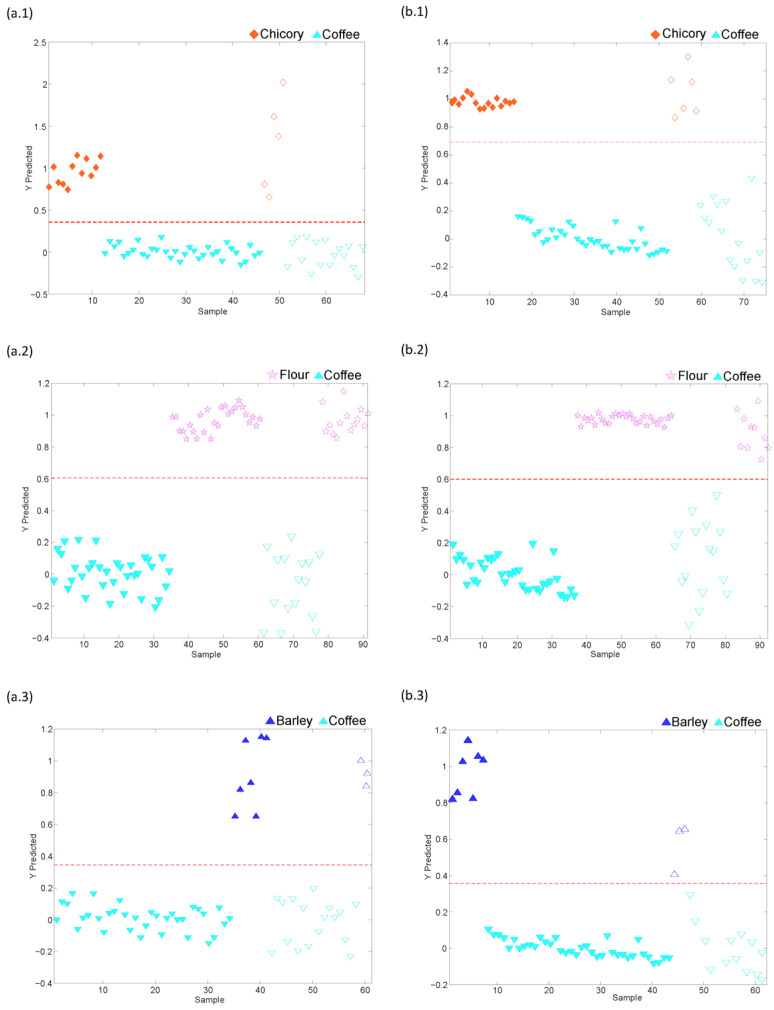
Paired PLS-DA score plots of Y predictions vs. samples for the data obtained using the (**a**) FIA-MS method and (**b**) LC-MS method to evaluate (**1**) coffee vs. chicory, (**2**) coffee vs. flour, and (**3**) coffee vs. barley. Filled and empty symbols correspond to calibration and prediction sets, respectively. Red lines represent the threshold between classes.

For each case, 70% of the samples from both classes were randomly selected to construct the calibration model, while the remaining 30% were reserved for prediction. The number of LVs and the values of the sensitivity, specificity, and classification errors for both calibration and prediction models are summarized in Table 1. Additionally, Figure 4 displays representative paired PLS-DA prediction plots. The sample number illustrates the clear class separation achieved using both the FIA-MS and LC-MS datasets for all coffee vs. adulterant (chicory, flour, barley) comparisons. Similar plots for the classification by coffee variety and geographical origin region are shown in the Supplementary Materials (Figure S4).

As reported in Table 1, the classification performance was outstanding across all models. In the paired comparisons between coffee and each adulterant (chicory, flour, barley), the FIA-MS method achieved 100% sensitivity and specificity in both calibration and prediction sets, resulting in 0% classification errors. The LC-MS method also delivered an excellent performance, with perfect classification in most cases. Minor decreases were observed in the prediction metrics for the coffee vs. flour and coffee vs. barley, with sensitivity and specificity above 90% and classification errors below 5%. This slight decrease in the classification performance for the LC-MS method could be attributed to partial overlap in the chemical profiles of some adulterants (e.g., flour and barley) with coffee. This overlap may be related to common roasting-derived compounds or matrix effects, which can reduce the discrimination capacity of the LC-MS fingerprinting in certain binary comparisons. In contrast, the FIA-MS method achieved perfect classification in both comparisons, with 0% classification errors. These results suggest that, although LC-MS may offer slightly enhanced resolution for chemically complex or overlapping samples due to chromatographic separation, in this study, FIA-MS actually yielded an equal or even superior classification performance in these specific cases.

Furthermore, as Table 1 shows, the classifications according to the coffee variety and coffee geographical origin for both methods show 100% sensitivity and specificity values. These results confirm the high discriminatory capability of the chemical fingerprints obtained by both FIA-MS and LC-MS. The consistent performance across all the studied cases highlights the potential of these non-targeted approaches for the rapid, reliable authentication of coffee samples, even in the presence of adulterants.

It is worth noting that the present study employed the same type of coffee and adulterant samples as those used in previous works where PLS-DA paired models were developed using non-targeted HPLC-UV/FLD fingerprinting [14] and HS-SPME-GC-MS [17] methodologies. This allows for a direct comparison of the results across different analytical platforms.

For instance, Núñez et al. [14] validated HPLC-UV and HPLC-FLD fingerprinting strategies using paired PLS-DA to classify coffee against chicory, flour, and barley. Perfect classification (100% sensitivity and specificity) was achieved in both calibration and validation sets across all comparisons. However, FLD-based models exhibited minor sample dispersion, particularly in the coffee versus barley comparison. The chromatographic runs required over 40 min per sample in the HPLC-UV and HPLC-FLD separations. In contrast, the FIA-MS paired models demonstrated an equivalent classification performance, achieving 100% sensitivity and specificity in all cases, including the most challenging comparisons (e.g., coffee vs. barley).

The classification results obtained using the paired PLS-DA models based on GC–MS fingerprinting [17] also demonstrate the effectiveness for coffee authentication according to sample origin and variety. The sensitivity and specificity values were consistently high, often reaching 100% for calibration and prediction. In comparison, the paired models developed in this study using FIA-MS exhibited equivalent classification performances across all evaluated scenarios.

These findings highlight FIA-MS as a highly efficient and reliable alternative to chromatographic fingerprinting techniques for coffee authentication. The method combines rapid analysis with robust classification capabilities, offering significant advantages for high-throughput screening and routine quality control applications in the coffee industry.

### 3.5. PLS Regression for Coffee Adulteration Detection and Quantification

PLS regression was applied to evaluate the capabilities of the developed non-targeted FIA-MS and LC-MS methodologies for detecting and quantifying coffee adulterations. As a proof of concept, Vietnamese Arabica coffee was used as the base matrix, and three common coffee substitutes—chicory, flour, and barley—were studied because of their potential economic and sensory impacts on the final product quality.

Three adulteration cases were assessed independently. As shown in Table S2 (Supplementary Materials), calibration models were built using a range of adulteration levels (0–100%), while prediction models were evaluated at intermediate levels (15–85%). More design details are presented in Section 2.2.

Table 2 summarizes the PLS regression results for the three FIA-MS and three LC-MS datasets.

FIA-MS yielded the lowest calibration errors (<0.91%) and prediction errors, ranging from 6.97% to 11.70%, depending on the adulterant. LC-MS also demonstrated a good performance, with calibration errors between 0.43% and 1.77% and prediction errors below 9.59%.

Although both platforms showed remarkable predictive capabilities, FIA-MS achieved slightly lower calibration errors overall, while LC-MS provided improved prediction accuracy in some cases. These findings confirm the suitability of both non-targeted approaches for quantitative adulteration assessment. The acceptable error ranges support their use in the routine screening and quality control of coffee authenticity, particularly where FIA-MS offers advantages in speed and cost-effectiveness.

A similar design was previously studied employing the HPLC-UV and HPLC-FLD non-targeted methods by Núñez et al. [14]. Compared with the current results, the calibration and prediction errors were consistently low, with values below 1.4% and 1.9% for HPLC-UV, respectively, and below 0.5% and 2.2% for HPLC-FLD, respectively, in quantifying chicory, barley, and wheat flour in Vietnamese Arabica coffee adulterations. FIA-MS data yielded calibration and prediction errors below 0.91% and 11.7%, respectively, confirming an acceptable quantitation. As a result, it is important to consider the substantial advantages offered by FIA-MS as a rapid, high-throughput, and cost-effective alternative for coffee adulteration screening. These benefits make FIA-MS particularly attractive for routine quality control, where speed and operational simplicity are essential.

### 3.6. FIA-MS and LC-MS Greenness and Blueness Evaluation

**Figure 5:**
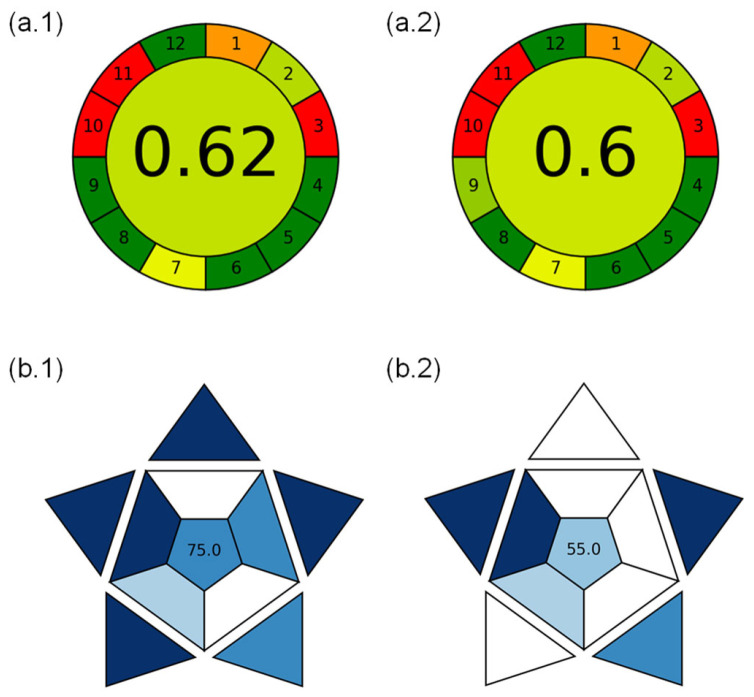
Greenness and blueness evaluation of the proposed FIA-MS and LC-MS methods. (**a**) Circular diagram generated with the Analytical Greenness Calculator v.0.4 based on the AGREE metric, and (**b**) molecular-like diagram obtained from BAGI beta 0.9 software, for (**1**) FIA-MS and (**2**) LC-MS in both cases.

To provide a comprehensive perspective on the analytical suitability of the proposed methods for coffee classification and authentication, both the environmental sustainability and operational applicability were systematically evaluated (Figure 5). The AGREE (Analytical GREEnness Metric Approach) and BAGI (Blue Applicability Grade Index) tools were employed to assess the greenness and blueness scores.

In Figure 5, the colors used in the greenness assessment (red, yellow, green, etc.) represent the performance of each individual criterion, where green indicates higher compliance with sustainability principles and red indicates lower compliance. For the blueness assessment, the shades of blue correspond to the scores of digitalization-related criteria, with darker blue representing a higher performance. This color coding allows for an intuitive visualization of the performance of the method in terms of environmental and digitalization metrics.

The AGREE methodology, implemented via Analytical Greenness Calculator v.0.4 software [26], evaluated the methods against the 12 principles of Green Analytical Chemistry (GAC). Figure 5(a.1),(a.2) show the FIA-MS and LC-MS scores across multiple domains (0.62 and 0.6, respectively, with 1 being the maximum score), including low sample consumption, lack of derivatization, reduced number of preparation steps, and minimal generation of analytical waste. These characteristics reflect the well-established strengths of MS-based techniques when aligned with green chemistry principles. FIA-MS demonstrated a considerably higher sample throughput, achieving approximately 30 samples per hour, in contrast to ~1 sample/hour for LC-MS. This significantly improves its energy efficiency and reduces the environmental burden per analysis. Despite these strengths, no method supports in situ measurements (both being laboratory-based and off-line). Nevertheless, the overall AGREE profiles reflect a good environmental performance, with FIA-MS standing out as the greener alternative due to its higher efficiency and lower per-sample resource demand. While FIA-MS demonstrates several environmentally friendly aspects, its AGREE score of 0.62 indicates moderate sustainability rather than an excellent level. Therefore, the method should be considered relatively sustainable, with room for improvement in green metrics. The explained criteria for the FIA-MS and LC-MS greenness evaluation are in Table S4 (Supplementary Materials).

To assess the practical feasibility of implementing these techniques in routine laboratory workflows, the blueness was evaluated through the BAGI software (beta version 0.9) by Manousi et al. [27]. This tool provides a multi-parametric evaluation based on ten distinct operational criteria. Figures 5(b.1) and 5(b.2) show that FIA-MS and LC-MS achieved high scores across multiple domains (75 and 55, respectively, with 100 being the maximum score for this evaluation).

Both FIA-MS and LC-MS delivered multi-analyte qualitative information for more than 15 compounds and relied on advanced instrumentation and commercially available reagents. However, important differences influenced the overall applicability scores. FIA-MS demonstrated superior throughput. In terms of automation, both methods were semi-automated systems with non-standard devices, incorporating autosamplers. Despite this, FIA-MS benefits from a reduced analytical time and streamlined operational workflow, ultimately enhancing its practical utility. Cumulatively, the BAGI results indicated that FIA-MS is better suited for high-efficiency environments, where speed and minimal manual intervention are critical. In contrast, LC-MS may be more appropriate in targeted or low-throughput applications where detailed separation is prioritized over processing speed. Table S5 provides the BAGI criteria for FIA-MS and LC-MS.

## 4. Conclusions

This work presents a comprehensive evaluation of FIA-MS and LC-MS for the characterization, classification, and authentication of coffee samples in the context of food adulteration detection.

Through exploratory PCA, the inherent chemical differences among pure coffee and its most common adulterants (chicory, flour, and barley) were reflected in distinct clustering patterns for both employed methodologies. Furthermore, PCA also revealed consistent groupings of coffee samples according to their botanical variety (Arabica vs. Robusta) and geographical origin (Vietnam vs. Cambodia).

The application of supervised PLS-DA models demonstrated an excellent classification performance across all evaluated scenarios and for both the FIA-MS and LC-MS non-targeted methods. Global models built to separate all coffee from all adulterants (and different types of coffees from each other) yielded excellent sensitivity and specificity values, with classification errors below 5% in most cases. Moreover, successful discrimination between coffee varieties and origins was also achieved.

From a comparative standpoint, LC-MS offered a slightly superior classification accuracy, especially when dealing with chemically similar classes, due to the added benefit of chromatographic separation. This advantage was particularly evident in the classification of origin and subtle adulterations, where the complexity of the matrix required finer signal discrimination. However, FIA-MS proved to be a remarkably efficient alternative, delivering excellent classification metrics in only 1.5 min per sample, making it highly attractive for rapid, high-throughput screening workflows.

Furthermore, compared to our previous fingerprinting study based on HPLC-UV-FLD detection, the implementation of FIA-MS enables the acquisition of richer and more informative spectral profiles. The use of full-scan mass spectrometry provides access to a broader range of compounds, thereby offering an enhanced chemical dimensionality essential for untargeted classification. In addition, the direct-injection FIA-MS workflow significantly reduces the analysis time, allowing for the high-throughput screening of coffee samples in a fraction of the time required for conventional HPLC-UV-FLD methods.

The AGREE and BAGI evaluation revealed clear contrasts between the two tested methodologies. FIA-MS obtained an AGREE score of 0.62 and a BAGI score of 75/100, reflecting a favorable environmental profile and strong practical performance, particularly due to its high sample throughput. In comparison, LC-MS achieved slightly lower scores, with an AGREE value of 0.60 and a BAGI score of 55/100, mainly limited by its slower workflow. The results indicate that FIA-MS is the more sustainable and versatile option for implementation in routine food authentication protocols. These findings reinforce the utility of greenness and blueness metrics as decision-making tools in the development and selection of modern analytical approaches. LC-MS is optimal for confirmatory analysis requiring more resolution and accuracy, while FIA-MS offers a faster and more scalable approach for routine or preliminary screening applications. The successful implementation of both methods reinforces the value of non-targeted strategies in food authentication.

In summary, this study demonstrates that a strategy based on FIA-MS fingerprinting combined with chemometric analysis offers a rapid, reliable, and relatively environmentally friendly approach for coffee authentication and adulteration detection. While LC-MS remains the optimal option for confirmatory testing, FIA-MS achieves a comparable classification accuracy in most scenarios, with the added advantages of faster analysis and higher throughput. This makes it especially suitable for routine screening and large-scale monitoring applications in the coffee industry.

## Figures and Tables

**Table 1:** LVs and sensitivity, specificity, and classification error values obtained for calibration and prediction on paired PLS-DA models when studying the classifications of the analyzed samples by the FIA-MS and LC-MS non-targeted methods.

			Calibration			Prediction		
Method	LVs	Class	Sensitivity (%)	Specificity (%)	Classification Error (%)	Sensitivity (%)	Specificity (%)	Classification Error (%)
Coffees vs. Chicory								
**FIA-MS**	2	Coffee	100	100	0	100	100	0
		Chicory	100	100	0	100	100	0
**LC-MS**	2	Coffee	100	100	0	100	100	0
		Chicory	100	100	0	100	100	0
Coffee vs. Flour								
**FIA-MS**	2	Coffee	100	100	0	100	100	0
		Flour	100	100	0	100	100	0
**LC-MS**	2	Coffee	100	100	0	100	90	5.0
		Flour	100	100	0	90	100	5.0
Coffee vs. Barley								
**FIA-MS**	2	Coffee	100	100	0	100	100	0
		Barley	100	100	0	100	100	0
**LC-MS**	2	Coffee	100	100	0	100	92.3	4.0
		Barley	100	100	0	92.3	100	4.0
Arabica Coffee vs. Robusta Coffee								
**FIA-MS**	4	Arabica	100	100	0	100	100	0
		Robusta	100	100	0	100	100	0
**LC-MS**	2	Arabica	100	100	0	100	100	0
		Robusta	100	100	0	100	100	0
Vietnamese Coffee vs. Cambodian Coffee								
**FIA-MS**	3	Vietnam	100	100	0	100	100	0
		Cambodia	100	100	0	100	100	0
**LC-MS**	2	Vietnam	100	100	0	100	100	0
		Cambodia	100	100	0	100	100	0

**Table 2:** Evaluation of the coffee adulteration cases by PLS using obtained FIA-MS and LC-MS data as sample chemical descriptors.

	Coffee	Adulterant	LVs	Calibration Errors (%)	Prediction Errors (%)
FIA-MS	Vietnamese Arabica Coffee	Chicory	4	0.31	6.97
		Flour	4	0.27	10.31
		Barley	4	0.91	11.7
LC-MS	Vietnamese Arabica Coffee	Chicory	3	0.43	9.15
		Flour	2	1.77	7.48
		Barley	4	0.52	9.59

## Data Availability

Data is available upon request to the authors.

## Supplementary Materials

The following supporting information can be downloaded at https://www.mdpi.com/article/10.3390/foods14172931/s1, Table S1: Description of the analyzed samples; Table S2. Coffee concentration levels used in the calibration and validation sets for each adulteration case, where X denotes the original coffee sample and Y represents the adulterant coffee sample; Table S3. LVs and sensitivity, specificity, and classification error values obtained by PLS-DA when studying the classifications of the analyzed samples with data obtained from the FIA-MS and LC-MS non-targeted methods; Table S4. Evaluation of the environmental sustainability of the proposed FIA-MS and LC-MS methods using the Analytical Greenness Calculator v.0.4 (2020), which is based on the AGREE (Analytical GREEnness Metric Approach) methodology developed by Pena-Pereira et al. (Pena-Pereira et al., 2020) [26]. Each of the twelve principles of Green Analytical Chemistry (GAC) is scored individually, and the rationale for each selected input is also provided; Table S5. Evaluation of the practical applicability of the proposed FIA-MS and LC-MS methods using the BAGI beta 0.9 software, following the methodology described by Manousi et al. (Manousi et al., 2023) [27]. The table includes the ten BAGI criteria, the selected input for each one, the corresponding score, and the justification for the choice; Figure S1. Non-targeted LC-MS fingerprints for selected (a) Vietnamese Arabica coffee, (b) Vietnamese Robusta coffee, (c) Cambodian coffee, (d) chicory, (e) flour, and (f) barley; Figure S2: PCA score plots obtained when (a) FIA-MS and (b) LC-MS fingerprints were used as sample chemical descriptors to study coffee samples according to their (1) variety (Arabica vs. Robusta) and (2) geographical production region (Vietnam vs. Cambodia); Figure S3: PLS-DA score plots obtained when (a) FIA-MS and (b) LC-MS fingerprints were used as sample chemical descriptors to study coffee samples according to their (1) variety (Arabica vs. Robusta) and (2) geographical production region (Vietnam vs. Cambodia); Figure S4: Paired PLS-DA score plots of Y predictions vs. samples for (a) FIA-MS- and (b) LC-MS-obtained fingerprints according to the (1) coffee variety (Arabica vs. Robusta) and (2) geographical production region (Vietnam vs. Cambodia).
