# Supplementary material for: An FIA-MS Method for Rapid Coffee Adulteration Detection: A Comparative Study with a Non-Targeted LC-MS Approach
Source: Foods. 2025 Aug 22;14(17):2931. doi: 10.3390/foods14172931 (PMC12427724; doi:10.3390/foods14172931)

foods MDPI

Supplementary Material

FIA-MS Method for Rapid Coffee Adulteration Detection: A Comparative Study with a Non-Targeted LC-MS Approach

Nerea Núñez [a,*], Javier Saurina [a,b] and Oscar Núñez [a,b,c]

[a] Department of Chemical Engineering and Analytical Chemistry, University of Barcelona. Martí i Franquès 1-11, E08028, Barcelona, Spain.
[b] Research Institute in Food Nutrition and Food Safety, University of Barcelona, Recinte Torribera, Av. Prat de la Riba 171, Edifici de Recerca (Gaudí), Santa Coloma de Gramenet, E08921 Barcelona, Spain.
[c] Serra Húnter Fellow, Departament de Recerca i Universitats, Generalitat de Catalunya, Via Laietana 2, E08003 Barcelona, Spain.
* Correspondence: Corresponding author: Nerea Núñez
Department of Chemical Engineering and Analytical Chemistry, University of Barcelona.
Martí i Franquès 1-11, E08028 Barcelona, Spain.
e-mail: nereant7@gmail.com

Table S1. Description of the analyzed samples.

Coffee			
**Sample Class**	**Sample Commercial Name**	**Sample Type**	**Number of Samples**
Coffee	Hoy An Roastery (Arabica)	Arabica Coffee	4
	Coc Soc Coffee (Arabica)		4
	M'JA (Arabica)		2
	An Phu (Arabica)		8
	Hoy An Roastery (Robusta)	Robusta Coffee	5
	Coc Soc Coffee (Robusta)		2
	M'JA (Robusta)		6
	An Phu (Robusta)		6
	-	Cambodian Coffee	6
Chicory	Lima Chicory	Chicory	7
	La Niña Chicory	Chicory	9
Flour	Moulin des Moines Wheat Flour	Wheat Flour	5
	ECO Sana Rice Flour	Rice Flour	4
	ECO Sana Rye Flour	Rye Flour	8
	El Granero Integral Cornmeal Flour	Cornmeal Flour	9
	El Granero Integral Rye Flour	Rye Flour	14
	El Granero Integral Wheat Flour	Wheat Flour	4
	El Granero Integral Oatmeal Flour	Oatmeal Flour	6
Barley	El Granero Integral Barley	Malta Barley	5
	El Granero Integral Pearl Barley	Pearl Barley	5

Table S2. Coffee concentration levels used in the calibration and validation sets for each adulteration case, where X denotes the original coffee sample and Y represents the adulterant coffee sample.

**Calibration Set**	**X%**	100	80	60	40	20	0
	**Y%**	0	20	40	60	80	100
**Validation Set**	**X%**	85	75	50	25	15	
	**Y%**	15	25	50	75	85	

Table S3. LVs and sensitivity, specificity, and classification error values obtained by PLS-DA when studying the classifications of the analyzed samples with data obtained from the FIA-MS and LC-MS non-targeted methods.

**Method**	**LVs**	**Class**	**Sensitivity (%)**	**Specificity (%)**	**Classification error (%)**
Coffee vs. Adulterant Classification					
**FIA-MS**	3	Coffee	100	100	0
		Chicory	87.5	100	0.06
		Flour	100	90.8	0.05
		Barley	87.5	100	0.06
**LC-MS**	3	Coffee	100	100	0
		Chicory	100	100	0
		Flour	100	100	0
		Barley	100	100	0
Arabica Coffee vs. Robusta Coffee Classification					
**FIA-MS**	3	Arabica	100	100	0
		Robusta	100	100	0
**LC-MS**	3	Arabica	100	100	0
		Robusta	100	100	0
Vietnamese Coffee vs. Cambodian Coffee Classification					
**FIA-MS**	3	Vietnam	100	100	0
		Cambodia	100	100	0
**LC-MS**	3	Vietnam	100	100	0
		Cambodia	100	100	0

Table S4. Evaluation of the environmental sustainability of the proposed FIA-MS and LC-MS methods using the Analytical Greenness Calculator v.0.4 (2020), which is based on the AGREE (Analytical GREEnness Metric Approach) methodology developed by Pena-Pereira et al. (Pena-Pereira et al., 2020) [26]. Each of the

twelve principles of Green Analytical Chemistry (GAC) is scored individually, and the rationale for each selected input is also provided.

	FIA-MS		LC-MS	
**Principle (GAC)**	**Selected Input**	**Justification**	**Selected Input**	**Justification**
1. Direct analytical techniques should be applied to avoid sample treatment.	Sample procedure: external sample pretreatment and treatment and batch analysis (reduced number of steps).	External sample preparation is required.	Sample procedure: external sample pretreatment and treatment and batch analysis (reduced number of steps).	External sample preparation is required.
2. Minimal sample size and minimal number of samples are goals.	Amount of sample: 1.0 g.	A low sample amount is used, reducing consumption and waste generation.	Amount of sample: 1.0 g.	A low sample amount is used, reducing consumption and waste generation.
3. If possible, measurements should be performed in situ.	Positioning of the analytical device: off-line.	The system does not allow in situ analysis, as it is a laboratory-based technique; however, the process is rapid.	Positioning of the analytical device: off-line.	The system does not allow in situ analysis, as it is a laboratory-based technique; however, the process is rapid.
4. Integration of analytical processes and operations saves energy and reduces the use of reagents.	Distinct steps in the sample preparation procedure: 3 or fewer.	The procedure is simple with few steps, reducing energy consumption and reagent use.	Distinct steps in the sample preparation procedure: 3 or fewer.	The procedure is simple with few steps, reducing energy consumption and reagent use.
5. Automated and miniaturized methods should be selected.	- Degree of automation: automatic.<br>- Sample preparation: none or miniaturized.	The system is automated and requires minimal sample preparation, improving efficiency and reproducibility.	- Degree of automation: automatic.<br>- Sample preparation: none or miniaturized.	The system is automated and requires minimal sample preparation, improving efficiency and reproducibility.
6. Derivatization should be avoided.	Not needed.	No derivatization is necessary.	Not needed.	No derivatization is necessary.
7. Generation of a large volume of analytical waste should be avoided, and proper management of analytical waste should be provided.	Low generated volume.	The volume of generated waste is low, complying with waste minimization principles.	Low generated volume.	The volume of generated waste is low, complying with waste minimization principles.
8. Multi-analyte or multi-parameter methods are preferred versus methods using one analyte at a time.	Spectral fingerprint.<br><br>Total of 30 samples analyzed per hour, approximately.	The technique allows for the simultaneous detection of multiple analytes.<br><br>Approximately 2 min per sample was needed for the analysis.	Spectral fingerprint.<br><br>One sample analyzed per hour, approximately.	The technique allows for the simultaneous detection of multiple analytes.<br><br>Approximately 40 min per sample was needed for the analysis.
9. The use of energy should be minimized.	Low energy per sample.	Energy consumption per sample is low due to automation and the rapid nature of the method.	Low energy per sample.	Energy consumption per sample is low due to automation and the rapid nature of the method.
10. Reagents obtained from renewable sources should be preferred.	None of the reagents are from bio-based sources.	The reagents employed are not bio-based.	None of the reagents are from bio-based sources.	The reagents employed are not bio-based.
11. Toxic reagents should be eliminated or replaced.	Solvents are employed.	The use of solvents, which may be toxic, indicates a limitation regarding safety and sustainability.	Solvents are employed.	The use of solvents, which may be toxic, indicates a limitation regarding safety and sustainability.
12. Operator's safety should be increased.	No hazards.	The method presents no significant hazards to the operator, ensuring safe working conditions.	No hazards.	The method presents no significant hazards to the operator, ensuring safe working conditions.

Table S5. Evaluation of the practical applicability of the proposed FIA-MS and LC-MS methods using the BAGI beta 0.9 software, following the methodology described by Manousi et al. (Manousi et al., 2023) [27]. The table includes the ten BAGI criteria, the selected input for each one, the corresponding score, and the justification for the choice.

	FIA-MS		LC-MS	
**Principle (BAGI criterion)**	**Selected Input**	**Justification**	**Selected Input**	**Justification**
1. Type of analysis	Qualitative	FIA-MS provides qualitative information through sample fingerprints.	Qualitative	FIA-MS provides qualitative information through sample fingerprints.
2. Multi- or single-element analysis	Multi-element analysis for > 15 compounds	The method allows for the simultaneous detection and analysis of more than 15 analytes in a single run.	Multi-element analysis for > 15 compounds	The method allows for the simultaneous detection and analysis of more than 15 analytes in a single run.
3. Analytical technique	Sophisticated instrumentation	FIA-MS requires advanced instrumentation combining flow injection analysis with mass spectrometry.	Sophisticated instrumentation	FIA-MS requires advanced instrumentation combining flow injection analysis with mass spectrometry.
4. Simultaneous sample preparation	One	Samples were prepared individually.	One	Samples were prepared individually.
5. Sample preparation	Multi-step sample preparation required	Sample treatment involves several steps, such as SPE extraction prior to analysis.	Multi-step sample preparation required	Sample treatment involves several steps, such as SPE extraction prior to analysis.
6. Samples per hour	> 10	The system can analyze more than 10 samples per hour.	One	The system can analyze one sample per hour, approximately.
7. Reagents and materials	Common commercially available reagents	The reagents used are standard and readily available commercially.	Common commercially available reagents	The reagents used are standard and readily available commercially.
8. Preconcentration	Sample treatment based on SPE extraction	Preconcentration of analytes is achieved by solid-phase extraction.	Sample treatment based in SPE extraction	Preconcentration of analytes is achieved by solid-phase extraction.
9. Degree of automation	Semi-automated with non-common devices	The method uses autosampler.	Semi-automated with non-common devices	The method uses autosampler.
10. Amount of sample	< 10 g food sample	The sample quantity used is less than 10 grams.	< 10 g food sample	The sample quantity used is less than 10 grams.

Figure S1. Non-targeted LC-MS fingerprints for selected (a) Vietnamese Arabica coffee, (b) Vietnamese Robusta coffee, (c) Cambodian coffee, (d) chicory, (e) flour, and (f) barley.

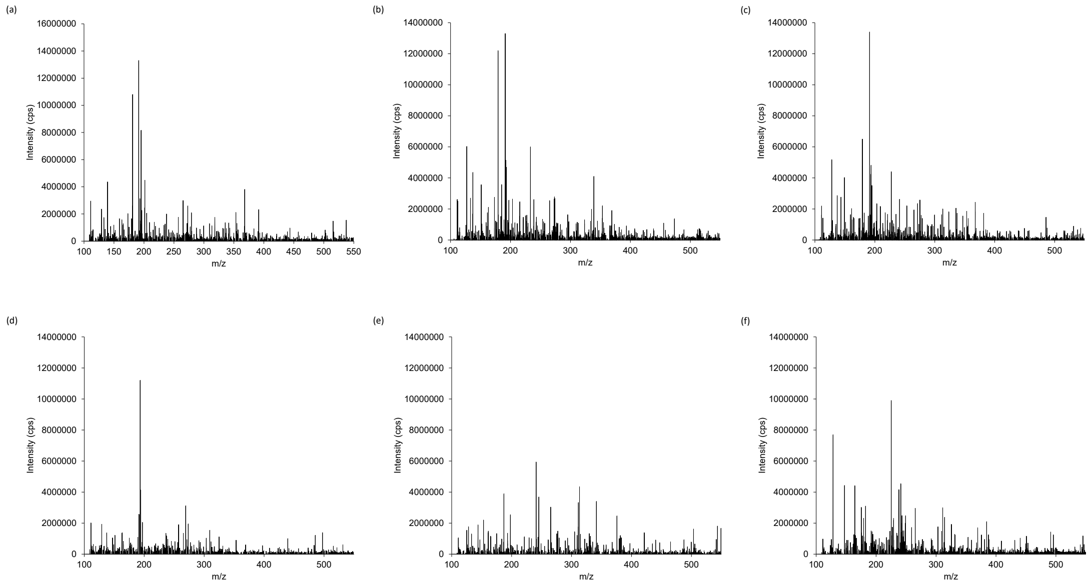

Figure S2. PCA score plots obtained when (a) FIA-MS and (b) LC-MS fingerprints were used as sample chemical descriptors to study coffee samples according to their (1) variety (Arabica vs. Robusta) and (2) geographical production region (Vietnam vs. Cambodia).

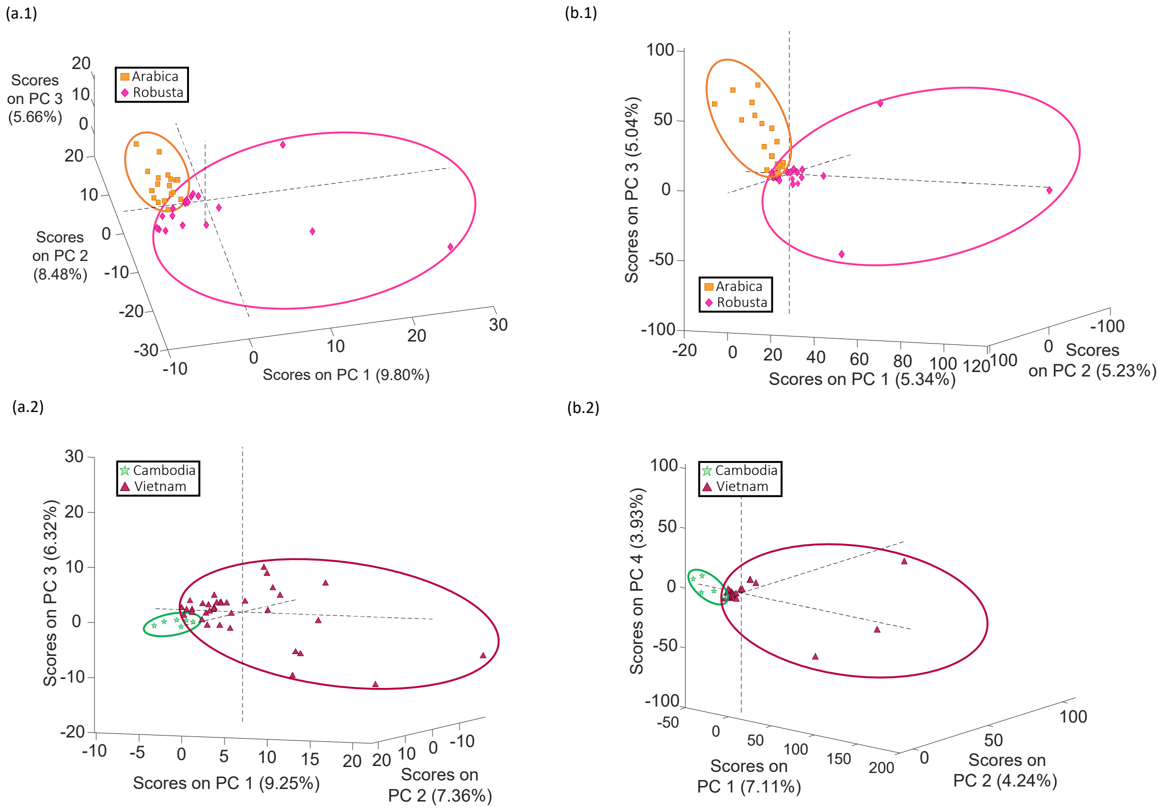

Figure S3. PLS-DA score plots obtained when (a) FIA-MS and (b) LC-MS fingerprints were used as sample chemical descriptors to study coffee samples according to their (1) variety (Arabica vs. Robusta) and (2) geographical production region (Vietnam vs. Cambodia).

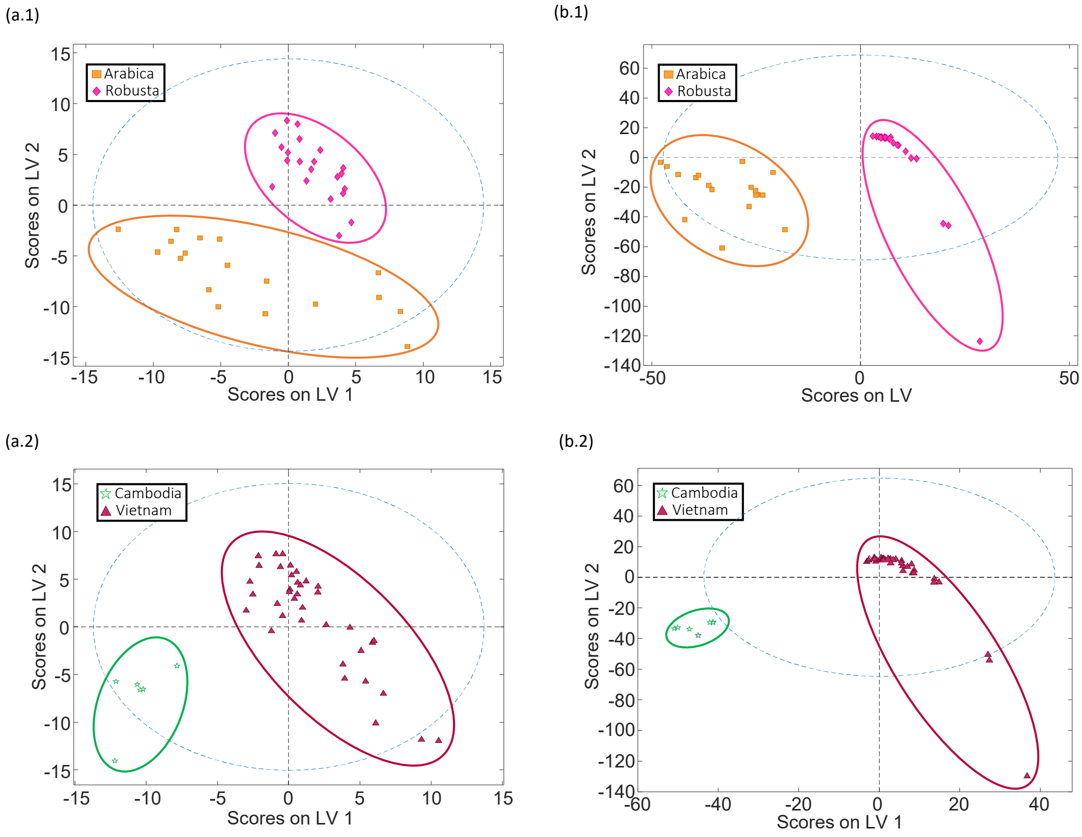

Figure S4. Paired PLS-DA score plots of Y predictions vs. samples for (a) FIA-MS- and (b) LC-MS-obtained fingerprints according to the (1) coffee variety (Arabica vs. Robusta) and (2) geographical production region (Vietnam vs. Cambodia).

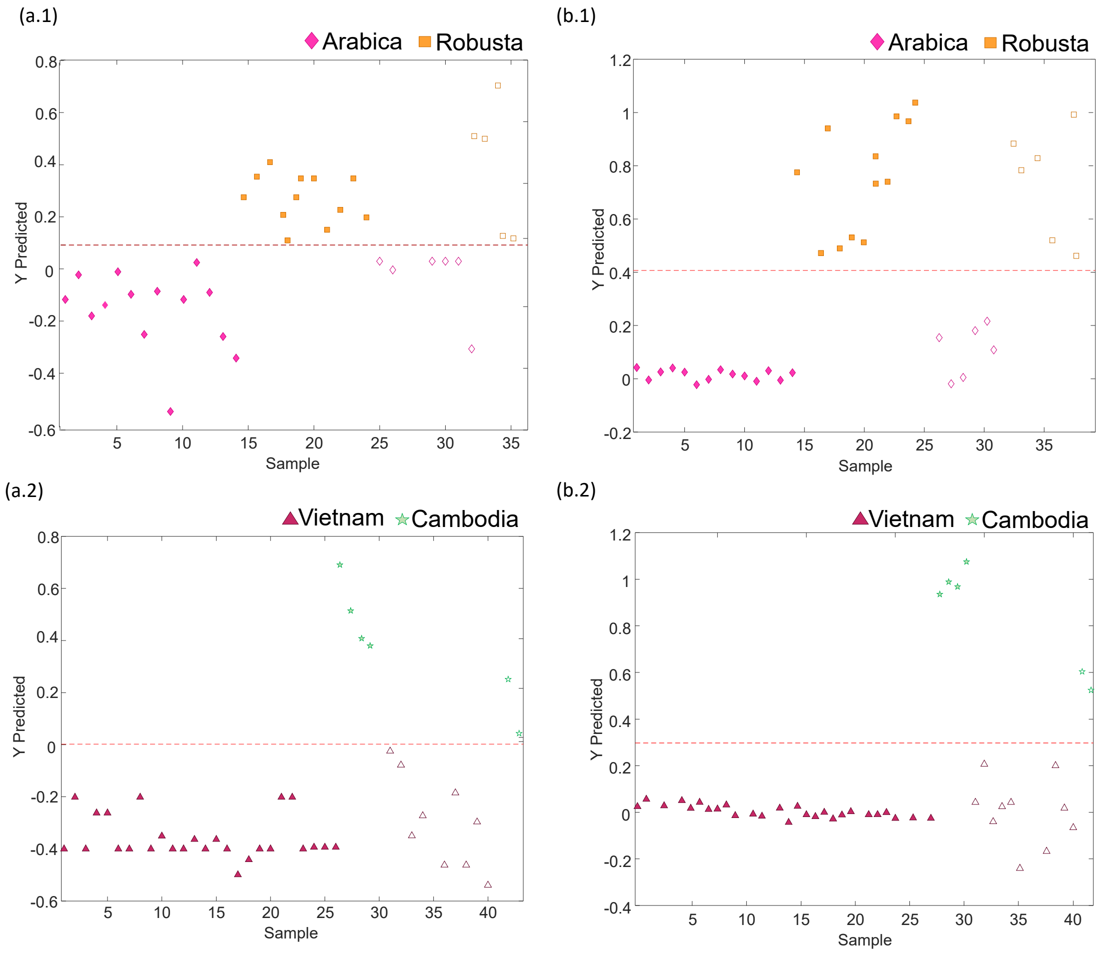

Supplement: Supplementary file 1 [file foods-14-02931-s001.zip › foods-3797105-supplementary.pdf]
